# Numerical Simulation of Thermal Conductivity of Foam Glass Based on the Steady-State Method

**DOI:** 10.3390/ma12010054

**Published:** 2018-12-24

**Authors:** Zipeng Qin, Gang Li, Yan Tian, Yuwei Ma, Pengfei Shen

**Affiliations:** 1 College of Water Conservancy and Architectural Engineering, Shihezi University, Shihezi 832000, China; qinzipeng18@mails.ucas.ac.cn (Z.Q.); tianyan1987610@163.com (Y.T.); myw819@shzu.edu.cn (Y.M.); spf2829879128@gmail.com (P.S.); 2 State Key Laboratory of Frozen Soil Engineering, Northwest Institute of Eco-Environment and Resources, Chinese Academy of Sciences, Lanzhou 730000, China; 3 College of Engineering Sciences, University of Chinese Academy of Sciences, Beijing 100049, China

**Keywords:** porous structure, numerical simulation, thermal conductivity, steady state method, foam glass

## Abstract

The effects of fly ash, sodium carbonate content, foaming temperature and foaming time on foam glass aperture sizes and their distribution were analyzed by the orthogonal experimental design. Results from the steady-state method showed a normal distribution of the number of apertures with change in average aperture, which ranges from 0.1 to 2.0 mm for more than 93% of apertures. For a given porosity, the thermal conductivity decreases with the increase of the aperture size. The apertures in the sample have obvious effects in blocking the heat flow transmission: heat flow is quickly diverted to both sides when encountered with the aperture. When the thickness of the sample is constant, the thermal resistance of the foam glass sample increases with increasing porosity, leading to better thermal insulation. Furthermore, our results suggest that the more evenly distributed and orderly arranged the apertures are in the foam glass material, the larger the thermal resistance of the material and hence, the better the thermal insulation.

## 1. Introduction

Fly ash foam glass is a kind of light, porous material made by proportionally mixing fly ash and glass powder, as the main raw materials, with an appropriate amount of additives and then pouring the mixture into a special mold to form the body, which then undergoes preheating, melting, foaming, foam-stabilizing and annealing [1,2,3,4]. This type of material is attractive for its high mechanical strength, low thermal conductivity, non-combustion (A-grade flame-retardant material), high softening temperature, good thermal and chemical stability, good sound insulation effect, strong corrosion resistance and is insect-proof. Therefore, it is highly attractive to be used in thermal insulation materials, lightweight filling materials, sound-absorbing materials, and lightweight concrete aggregates in fields like construction, water conservancy and transportation [5,6,7,8,9,10,11,12,13].

The research of Bian et al. showed that the foaming agent (SiC) had a great influence on the mechanical properties of the material [14]. Chen et al. found that the sintered fly ash foam glass at 800 °C had excellent comprehensive properties [15]. Qin et al. considered that the amount of fly ash had great impact on the strength, apparent density, porosity and thermal conductivity of foam glass, while the foaming temperature and foaming time had more remarkable influence on the pore structure and porosity distribution of foam glass [16,17,18]. Qian et al. studied the influence of foaming temperature and amount of foaming agent on pore structure, apparent density and compressive strength of foam glass, and the results showed that the pore structure of foam glass become uniform and the apparent density decreased in a certain range with the increase of firing temperature [19]. The research results of Tian et al. showed that insufficient cooling rate in the process of rapid expansion could not make the surface tension and viscosity of glass melt react with each other, which did not match the glass melt and gas expansion or contraction and resulted in foam glass surface depression [20]. Zhou et al. found that the pore size of the foamed glass increases with the increase of the sintering temperature, increases first and then decreases with the increase of the content of aluminum nitride, and it is easy to form large pores and connected pores in the products with the increasing temperature and the increasing foaming agent content [21,22]. Jurczyk et al. investigated the effectiveness of nanostructured titanium-10 wt% 45S5 Bioglass-1 wt% Ag composite foams as a novel class of antibacterial materials for medical applications. It was found that 1:1 Ti-10 wt% 45S5 Bioglass-1 wt% Ag/sugar ratio leads to porosities of about 70% with pore diameter of about 0.3–1.1 mm [23]. Je et al. studied the effect of interfacial bonding of glass hollow microspheres and a polymer matrix on the elastic properties of syntactic foam using representative volume element (RVE) models, and they also performed the finite element analysis to numerically estimate the elastic behavior of the models [24].

In this paper, relevant research on fly ash foam glass used as insulation material for building engineering was carried out. Fly ash foam glass, being porous, has a large number of apertures and hence, its thermal properties differ from those in denser materials. This property to material aspect has been extensively researched in the literature [25,26]. The aperture characteristic is one of the basic characteristics of porous materials. Most of the important properties of porous materials are directly or indirectly related to the aperture characteristic. Therefore, detailed characterization of the aperture structure of fly ash foam glass is crucial to the proper study on their materials properties.

The ratio of the raw materials, the amount of foaming agent, foaming temperature and foaming time are important parameters in the preparation of fly ash foam glass and, in turn, influences the size and distribution of the aperture structure in the foam glass. Moreover, these parameters are dependent on one another, as for instance if different raw materials or a different ratio of the raw materials are used, the amount of foaming agent, foaming temperature, and foaming time must vary to suitably prepare fly ash foam glass. In this work, the L_9_(3^4^) Orthogonal experiment was used to study the size and distribution of the aperture structures under various factors and the steady-state method was employed to numerically simulate the thermal conductivity of the foam glass. The effects of aperture size, glass thickness and porosity on the thermal conductivity were measured. This study provides insight into the design rationale of fly ash foam glass for manufacturing and engineering applications in building insulation materials.

## 2. Experiment 

### 2.1. Experimental Raw Materials

The fly ash used in the preparation of the foam glass was produced by Xinjiang Shihezi Tianye (Group) Thermal Power Plant. The glass powder came from discarded plate glass collected by a glass shop in Shihezi City, which was subsequently washed with clean water, fully dried, put into a tumbling mill machine for 2 hours and then passed through a 200-mesh sieve (Aperture 0.074 mm). The chemical composition and physical indicators of the fly ash and glass powder are shown in Table 1. The materials used were as follows: sodium carbonate (Na_2_CO_3_) as the foaming agent (analytical reagent, 99.5%, Tianjin Zhiyuan Reagent Co., Ltd., Tianjin, China), trisodium phosphate (Na_3_PO_4_·12H_2_O) as the foam stabilizer (analytical reagent, 98.0%, Tianjin Zhiyuan Reagent Co., Ltd., Tianjin, China), and boron nitride (BN) as the releasing agent (Model: JD-3028AAA, Dongguan Jiadan Lubricant Co., Ltd., Dongguan, China).

### 2.2. Sample Preparation

The fly ash, glass powder and trisodium phosphate were weighed according to a certain ratio, placed in a cement mortar mixer and stirred for 2~3 min, and then the prepared sodium carbonate solution was weighed and added into the mixer slowly. After stirring for 2~3 min, the mixture was put in a mold and placed on a concrete vibrating table for about 3 min, and then dried in an oven to obtain a foam glass rough-body. In order to heat the rough-body uniformly, refractory sand with thickness 1~1.5 cm was laid on the bottom of the resistance furnace chamber. The temperature control during the sinter process is mainly divided into four stages: the preheating stage: heat the rough-body from room temperature to 400 °C by 5 °C/min and dwell for 30 min to remove the immobilized and adsorbed water in the raw materials to eliminate the impact of the vapor produced in the heating process on the foaming stage. The foaming stage: the temperature was increased to 860 °C by 10 °C/min, and foaming was continued for 30 min to facilitate the chemical reactions through the use of the foaming agent, generating a large number of bubbles. The steady foaming and annealing stage: cool down to 600 °C at the rate of 15 °C/min, and dwell for 30 min so that the formed bubbles can stabilize quickly and the aperture structure at the time of completion in foaming can be maintained. Lastly, turn off the furnace, let the sample cool to ambient temperature and take it out of the furnace.

### 2.3. Analysis Instruments

Occhio Scan 600 is a product of a professional image-based particle size analysis instrument company, Occhio, Belgium. It has a powerful hardware design and particle graphic statistical processing capability and can manage multiple tasks like collecting images of tens of thousands of particles and statistical processing in a few minutes. By using this software, we are able to observe the aperture morphology, distribution and aperture wall thickness very clearly in the profile of the aperture structure on the 10mm × 20mm area of the geometric center of the sample section. The particle size analysis software Nano Measurer 1.2 was used to do the statistical analysis on the aperture size. Nano Measurer 1.2 is a simple and practical particle size analysis tool that can calibrate and measure various sizes of apertures. We use this software to calibrate the size of the apertures and obtain the average aperture. The average aperture is obtained by calculating the average value of the two calibrated apertures. Since the apertures are typically not standard circles, the average aperture is obtained by calibrating one aperture to a pore size of maximum diameter and the other to the minimum diameter. Thermal conductivity measurements were performed on the JTRG-III heat-flux-meter type thermal conductivity meter (Beijing Centurycom Environmental Technology Co., Ltd., Beijing, China). The thermal conductivity of the foam glass in our experiment was determined using a JTRG-III heat-flux-meter type thermal conductivity meter. This instrument adopts a single test piece double heat flow meter method. In order to ensure high precision, the samples were cut and flattened by a special cutter. Vaseline was applied on the upper and lower faces of the samples to make sure good contact between the upper and lower faces of the samples and the instrument. The foam glass was fully dried in the oven for 24 hours and then sealed in plastic bags and stored in the lab until measurements were run. The lab environment remained essentially the same and thus did not influence the measurements. The thermal conductivity of each set of samples was taken from the average thermal conductivity for three identical subsets of the sample. The measurements continued until the measured heat flow of the hot plate and the cold plate did not change appreciably, reaching a steady state at which point the measurements were stopped. Under normal circumstances for measurements of the glass foam samples, it took about 2 h for the measured heat flow to reach a steady state. Porosity refers to the percentage of pore volume in the material over the total volume. This measurement uses the apparent density of the test sample and the density in the dense state, thus the porosity of the material can be calculated by Equation (1) [27]:

(1) $$P=\frac{V_{a}}{V}=1-\frac{V_{s}}{V}=1-\frac{\rho_{s}}{\rho }$$

where *P* is the porosity, recorded as a percentage, $V_{a}$ is the volume of the pores in cm^3^, $V_{s}$ is the volume of the material in its dense state in units of cm^3^, $\rho_{s}$ is the density in the dense state with units of g/cm^3^, which was measured using the density of the dry powders in our experiments, and ρ is the apparent density of the material, including the pore volume, in units of g/cm^3^. In our experiment, three samples were selected and cut into regular blocks. For each sample, the length, width and height of the sample were measured, the volume, V, was calculated, and the mass, $G_{0}$, after drying (precision to 0.1 g) was weighed. The apparent density was calculated according to Equation (2). The apparent density of each set of samples was obtained by averaging the apparent density calculated for three identical subsets of the same sample (precision to 1 g/cm^3^).

(2) $$\rho =\frac{G_{0}}{V}$$

In Equation (2), $G_{0}$ represents the mass of the dry sample in grams and *V* is the apparent volume of the sample in units of cm^3^.

### 2.4. Analysis Instruments

Orthogonal experiment design is used to study multi-factor and multi-level methods. According to the orthogonality, some representative factors can be selected from the experiment for testing [28]. These representative factors are characterized by features like uniformly dispersed, neat and comparable. When three or more factors are involved in the experiments, and there may be interactions between the factors, the test workload will become very large and difficult to implement [29]. Orthogonal experiment design can be carried out using the orthogonal table method under the circumstances of knowing the number of factors, the number of factors’ levels, and whether there is interaction between different factors [30]. By using this method, it can achieve the equivalent results of implementing a large number of comprehensive tests.

The orthogonal table is a set of rules design tables, where L is the code of the orthogonal table, n is the number of trials, t is the number of levels, c is the number of factors, and the symbol for orthogonal table is expressed as L_n_(t^c^) [31]. For example, L_9_(3^4^) indicates that nine experiments are required, and up to four factors can be observed, each of which is three levels. In each column, the number of occurrences for different variables is equal. As in the three-level orthogonal table, any column will have a "1", "2", or "3", and the number of occurrences for these variables in any column is equal. In the case of 3 levels, there are 9 kinds of ordered pairs in any two columns (in the same horizontal row): (1,1), (1,2), (1,3), (2,1), (2,2), (2, 3), (3, 1), (3, 2), (3, 3), and the number of occurrences for each pair is also equal. The abovementioned embodies the features of the orthogonal table like uniformly dispersed, neat and comparable. In other words, each level of each factor pairs with each level of another factor, which is orthogonal.

As the basic raw material of foam glass, fly ash has a great influence on the strength, thermal conductivity and aperture structure of the material. The dosage of fly ash is usually controlled at 20%~30%. Sodium carbonate is a common foaming agent for preparing foam glass. At about 720 °C, it starts to react with SiO_2_ in the mixture to release CO_2_ gas. According to preliminary results, usually 2% to 6% of sodium carbonate is good enough for the foaming requirements. Because of the big impact the foaming temperature has on the aperture size and distribution, setting a reasonable foaming temperature is crucial to form apertures of appropriate sizes and with uniform distribution, thereby reducing the generation of aperture-connects and micro-apertures. Typically, different raw material compositions require different foaming temperatures. In our experiments, we chose 850~870 °C as our foaming temperature range. Foaming time is also an important factor that affects the aperture structure and the uniformity of foam glass material. If the foaming time is too short, the foaming agent will not be able to fully react, and thus the generated apertures will be too small and will not be uniformly-distributed; if the foaming time is too long, it will be easy to form micro-apertures and aperture-connects inside the material. Therefore, the foaming time is controlled at 20~30 min.

Fly ash content, sodium carbonate content, foaming temperature and foaming time were selected as the main factors F, C, T and S, respectively, and thermal conductivity and porosity were used as the test indexes. The L_9_(3^4^) Orthogonal experiment factors and levels are as shown in Table 2.

### 2.5. The L_9_(3^4^) Orthogonal Experiment Results

The L_9_(3^4^) Orthogonal experiment results are shown in Table 3.

### 2.6. Aperture Structure Analysis

**Figure 1:**
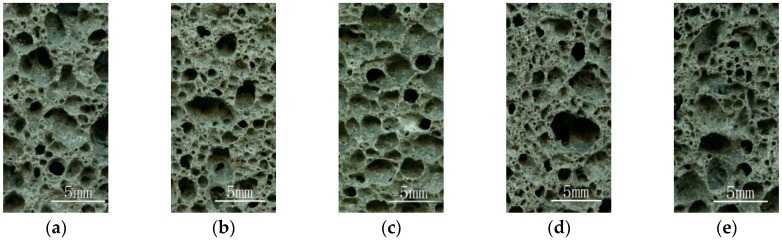

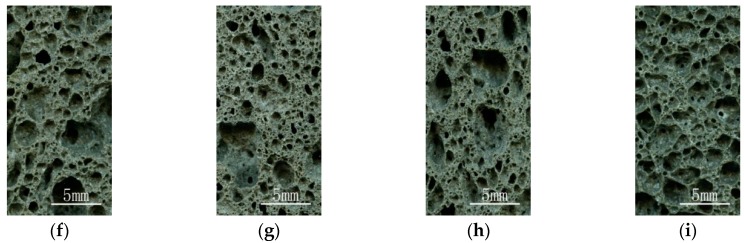
Aperture structure images of the foam glass samples, which are correspondingly numbered (**a**) Z1, (**b**) Z2, (**c**) Z3, (**d**) Z4, (**e**) Z5, (**f**) Z6, (**g**) Z7, (**h**) Z8 and (**i**) Z9, respectively.

The sample was cut along the longitudinal direction with an angle grinder, and the Occhio Scan 600 instrument (made by Occhio Instruments Company, Belgium) was used to scan the aperture structure (area of 10 mm × 20 mm, centered at the geometric center) of nine samples to obtain a sectional view of the sample, as shown in Figure 1.

As can be seen from Figure 1, the aperture structures of these nine groups are quite different, indicating that sodium carbonate and fly ash content, foaming temperature and time have a significant impact on the aperture structure. When foaming temperature is 850~860 °C, sodium carbonate content is 2~4%, foaming time is 20~25 min, the aperture walls of the foam glass samples Z1 and Z2 are thicker, the number of the apertures is smaller, and the aperture structure is relatively uniformly distributed. When the sodium carbonate content, foaming temperature and foaming time are increased, the aperture walls of foam glass samples Z3~Z9 gradually become thinner and the phenomenon of aperture-connecting becomes more obvious. When the foaming time reaches 30 min, the foaming temperature and the sodium carbonate content increases, leading to a significantly larger number of aperture-connects in the foam glass samples (Z4, Z8). Conversely, if the foaming temperature and the content of sodium carbonate are reduced, the number of the aperture-connects decreases.

**Figure 2:**
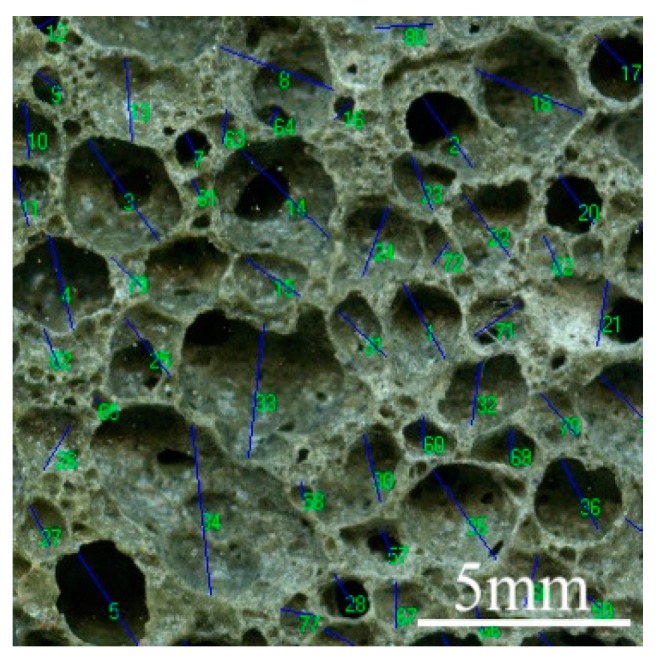
Statistical analysis on aperture size of foam glass samples.

The aperture size of the sample has been identified by using the Nano Measurer 1.2 particle size analysis software, shown in Figure 2.

The aperture sizes are divided into six interval grades: d ≥ 3 mm, 2 mm ≤ d < 3 mm, 1 mm ≤ d < 2 mm, 0.5 mm ≤ d < 1 mm, 0.1 mm ≤ d < 0.5 mm, and 0.01 mm ≤ d < 0.1 mm. The number of apertures in each interval was collected statistically, and the total aperture number, average aperture, and the standard deviation of the aperture were calculated, as shown in Table 4.

**Figure 3:**
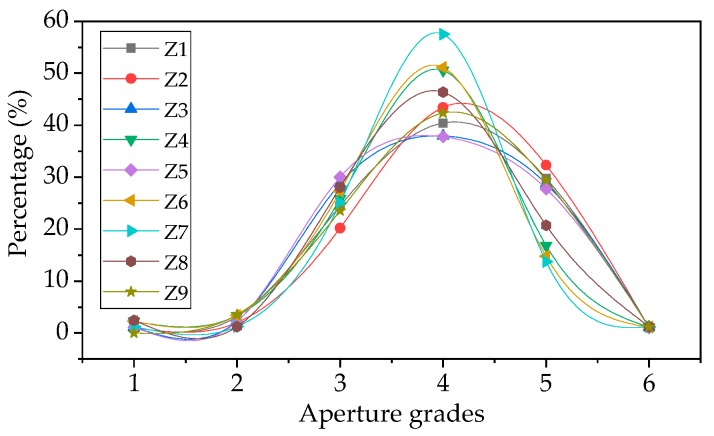
Diagram of aperture distribution in foam glass samples.

Using the data in Table 4, the relationship between the aperture grades and the corresponding aperture number in percentage are plotted in Figure 3.

Evidently from Figure 3, the aperture of all samples in the 3, 4, and 5 grades is above 93%, indicating that the sample aperture size is mainly distributed in the range of 0.1–2.0 mm. Due to the random distribution of sodium carbonate particles in the sample, the distribution of apertures in the sample also has a certain degree of discreteness. Within the six interval grades, the percentage of the apertures presents an approximate normal distribution.

## 3. Numerical model

### 3.1. Conditional Assumptions

To facilitate analysis of the thermal conductivity of the foam glass, the following assumptions are made: the foam glass is an isotropic material, the boundary of the foam glass is adiabatic, meaning that no heat is dissipated at the boundary, and the temperature is linearly distributed along the heat flow direction.

Heat transfer is classified into numerous mechanisms, such as thermal conduction, thermal convection and thermal radiation. In the heat transfer process, typically there are either overlaps in mechanisms or concurrent mechanisms, one or two of which usually dominates. In the modeling analysis, considering that the size of the apertures is very small, it can be assumed that no thermal convection occurs inside the apertures. During the thermal conductivity measurement process, the hot plate above the foam glass is heated to 45 °C, the lower cold plate is heated to 10 °C, and the laboratory ambient temperature is about 25 °C. Under these circumstances, the upper hot plate will produce some thermal radiation to cause the temperature of the upper surface of the foam glass to increase, but due to the excellent temperature control ability of the thermal conductivity device, the temperature of the upper surface of the foam glass can still retain constant. Thus, thermal radiation won’t exert much impact on the heat transfer between the upper and lower plates of the foam glass. Therefore, for the thermal analysis of foam glass, heat conduction is the only mechanism to be considered [32].

### 3.2. Model Establishment and Grid Generation

#### 3.2.1. The Establishment of a Geometric Model

To conduct numerical analysis on the foam glass so as to determine the shape, radius and the proportion of the apertures, the side lengths of the foam glass model were set as H and L, separately. The generation algorithm of the aperture geometry model is as follows:

- First bullet Determine the coordinates ($X_{i}$, $Y_{i}$) and radius *R_i_* of the number *i* aperture by generating a random number.
- If the aperture does not overlap with the boundary, and it does not overlap with the aperture generated previously either, then calculate the temporary variable $as=\pi R_{i}^{2}$.
- Compare the cumulative aperture area Sumarea and the total area of apertures Smax. If Sumarea>Smax, stop generating the apertures.

**Figure 4:**
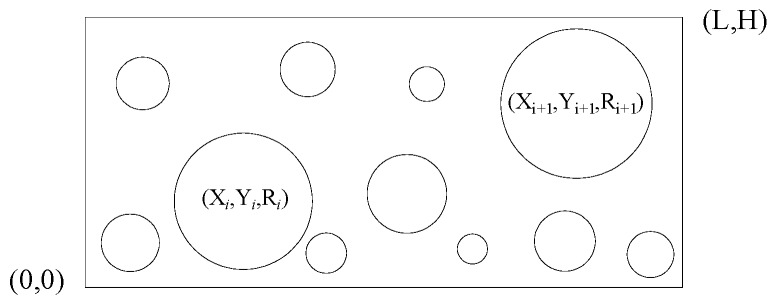
The generation of the aperture model.

In the aperture model generating process, the minimum radius and the maximum radius are controlled, while the position and size of each aperture are randomly generated. Whenever an aperture is generated, the position of the aperture within the rectangular region of side lengths H and L needs to be determined, that is, the positional relationship between the aperture and the four edges of the rectangle. For example, looking at Figure 4, suppose the parameters of the number i aperture are ($X_{i}$, $Y_{i}$*,* $R_{i}$) and the parameters of the qualified number *i*+1 aperture are ($X_{i+1}$, $Y_{i+1}$, $R_{i+1}$). If it satisfies the relationships of $X_{i}-R_{i}>X_{min}$ and $X_{i}+R_{i}>X_{max}$, then it indicates that the number *i* aperture is inside the region defined by the left and right boundaries. The position of the upper and lower boundaries of the number *i* aperture can be determined by the same method. When the number *i* aperture satisfies the upper, lower, left, and right boundary conditions, the positional relationship between the number *i* aperture and the other apertures satisfying the conditions inside the boundary can be determined [33].

The positional relationship between two apertures can be resolved by determining the relationship between the centers of the two aperture circles, the parameters of the apertures with satisfying conditions will then be saved in a predefined array library. Taking the number *i* and *i*+1 aperture as examples, if the parameters of the apertures satisfy:

(3) $$\sqrt{{\left(X_{i+1}-X_{i}\right)}^{2}-{\left(Y_{i+1}-Y_{i}\right)}^{2}}>R_{i+1}+R_{i}$$

It indicates that the two apertures do not overlap or intersect, that is, this aperture number i meets the requirements, and hence, the parameters of this aperture will be saved in the predefined array to calculate the aperture area. Lastly, Sumarea can be obtained by adding the areas of all the apertures satisfying the requirements. If the end condition (Sumarea>Smax) is satisfied, the aperture generation algorithm ends; otherwise, the above steps will be repeated.

**Figure 5:**
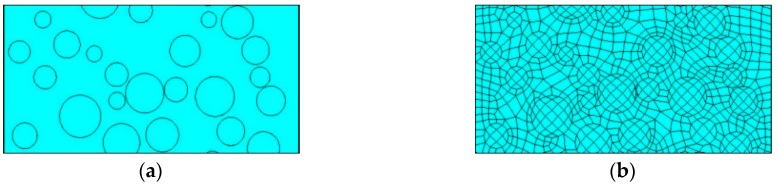
(**a**) Geometric model; (**b**) Meshing.

The area of the foam glass is 10 mm × 20 mm. Using the ANSYS (Large General Finite Element Analysis Software Developed by ANSYS Company) Parametric Design Language (APDL), with the porosity as a parameter, the aperture model of randomly-distributed apertures and controllable aperture sizes is established inside the rectangle. The two-dimensional numerical model of the foam glass is shown in Figure 5a.

#### 3.2.2. Define Material Properties

At room temperature, the thermal conductivity of foam glass is a constant. The thermal conductivity of air is 0.023 W/(m·K), and the thermal conductivity of the foam glass substrate (The foam glass substrate is a non-foamed foam glass material obtained by adding no sodium carbonate and trisodium phosphate without changing the ratio of the remaining raw materials) is 0.16 W/(m·K).

#### 3.2.3. Cell Selection and Meshing

Considering the porous feature of the foam glass, PLANE55 (a plane element or an axisymmetric ring element for two-dimensional heat conduction analysis), a two-dimensional thermal entity, was used. PLANE55 has a four-node structure and each node has only one degree of freedom on temperature, which can be used for two-dimensional heat conduction calculations. The geometric model meshing was undertaken by using the Target Surf meshing order, as shown in Figure 5.

### 3.3. Boundary Conditions

As mentioned in the conditional assumptions, the model is dominated by the heat conduction mechanism, ignoring the effects of heat convection and radiation. Its boundary conditions are: the temperature load of the left line is 45 °C, the convection load is applied to the designated right line, the ambient temperature is 10 °C, and the convection heat transfer coefficient is 25 W/(m^2^·K). The upper and lower boundaries of the foam glass model are adiabatic.

## 4. Results and Discussion

### 4.1. Model Validation

Heat was given according to the ANSYS post-processing results. The thermal conductivity, *λ*, can be related to the heat of the system. The mathematic expression of Fourier’s law for one-dimensional steady-state heat conduction is shown in Equation (4) [34]:

(4) $$\Phi =-\lambda A\frac{dT}{dy}$$

where Φ respresents the heat with units of W, λ is the thermal conductivity with units in W/(m·K), $\frac{dT}{dy}$ refers to the temperature change rate, and A is the heat transfer area with units of m^2^. According to Equation (4), we can get:

(5) $$\lambda =-\frac{\Phi }{A}\times \frac{dy}{dT}$$

For the one-dimensional steady-state heat conduction of the two-dimensional model, if the heat conduction length of the model is l and the model width is taken as b, then Equation (5) can be converted to Equation (6):

(6) $$\lambda =-\frac{\Phi }{l\times b}\times \frac{l}{\Delta T}$$

Namely,

(7) $$\lambda =-\frac{\Phi }{b\Delta T}$$

The value of the effective thermal conductivity of the model can be obtained by substituting the VALUE value (heat) and the temperature difference from the post-processing results into the above equation.

To verify the reliability of the model, with sample Z1 (porosity 30.54%, average aperture 0.65 mm) as the study objective, the thermal conductivities of the foam glass were measured using a steady-state heat flow meter; the numerical model was established under the same conditions. The simulation results were compared with the test results, shown in Table 5.

From Table 5, the simulated thermal conductivity of the foam glass is nearly identical to the measured value (relative error is less than 5.0%), which renders confidence in the simulations as a reliable model for the thermal conductivity of foam glass.

### 4.2. Effect of Aperture on Thermal Conductivity Performance of Foam Glass

**Figure 6:**
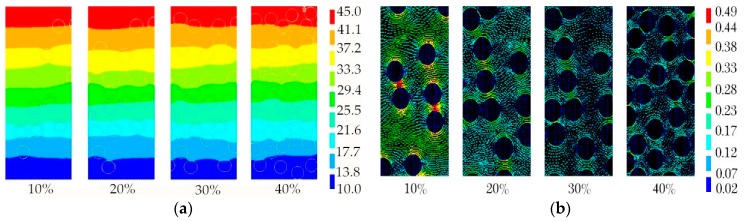
(**a**) Temperature nephogram; (**b**) Thermal vector nephogram.

The average aperture of foam glass samples is mostly distributed between 0.1~2.0 mm. The effect of aperture size on thermal conductivity of foam glass was explored for samples with aperture of 0.2 mm, 0.4 mm, 0.6 mm, 0.8 mm, 1.0 mm and 1.2 mm, and porosities of 10%, 20%, 30% and 40%. Temperature and thermal vector nephograms of the samples are shown in Figure 6.

**Figure 7:**
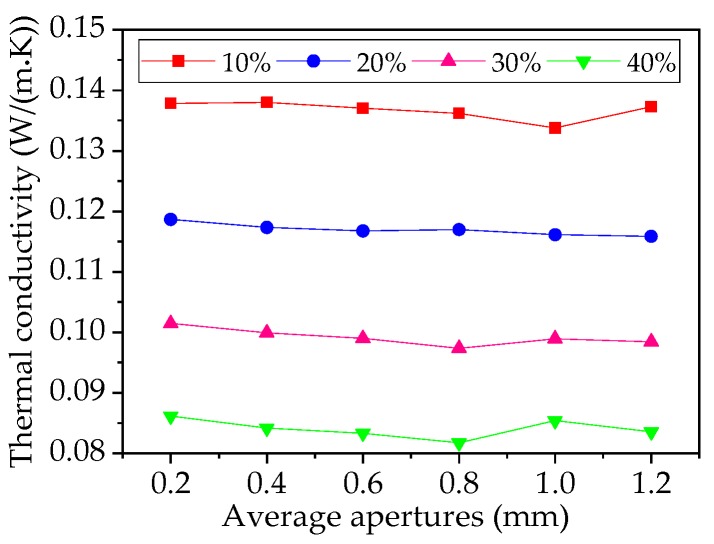
Relationship between average apertures and thermal conductivity at different porosity.

Evidently from Figure 6a, the temperature in the foam glass sample is distributed along the direction of heat flow from high to low, and this is more obvious at the boundary. As can be found from Figure 6b, the apertures in the foam glass sample play an essential role in blocking heat flow transmission; that is, when the heat flow meets the apertures, it diverts to the sides rapidly, transmitting along the back side of the aperture wall. Thus, there is essentially no heat flow into the apertures, and so the internal heat flow is minor. With the increase of porosity, the heat flow between apertures gradually decreases, which exemplifies that the apertures have a substantial impact on the thermal conductivity of foam glass. This relationship, that of thermal conductivity with average aperture at different porosities, is shown in Figure 7.

As can be seen from Figure 7, when the porosity increases, the thermal conductivity evidently decreases. When the porosity is kept constant, the thermal conductivity is also essentially constant as the aperture increases. The surface thermal conductivity of the abovementioned phenomenon is independent of the average aperture but is greatly affected by the porosity. Therefore, the thermal conductivity is mainly affected by the porosity rather than aperture size in the range of 0.2~1.2 mm as it shows in the diagram.

### 4.3. Effect of Thickness on Thermal Conductivity of Foam Glass

**Figure 8:**
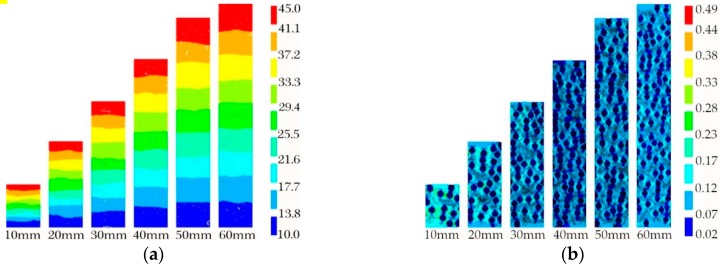
(**a**) Temperature nephogram; (**b**) Thermal vector nephogram.

The effect thickness has on thermal conductivity of foam glass was analyzed using a 0.8 mm aperture with varying sample thickness: 10 mm, 20 mm, 30 mm, 40 mm, 50 mm, and 60 mm. The temperature and thermal vector nephograms of the sample with varying thickness are shown in Figure 8.

As seen in Figure 8a, as the thickness of the foam glass sample is increased, the temperature range in the sample broadens, and the corresponding thermal insulation improves. The thermal vector nephogram (Figure 8b) demonstrates that the number and size of thermal resistance bands formed by the apertures decrease for foam glass thickness ranging from 10~30 mm. When the thickness of the sample is increased to 40 mm, the number of internal thermal resistance bands in the sample significantly increases, and the thermal-resistance effect is more pronounced. When the thickness of the sample is further increased to 50~60 mm, the length of the thermal resistance band has a greater influence on the thermal insulation of the foam glass. Therefore, the above analysis indicates that the thickness of the sample dictates the length of the thermal resistance band and the number of apertures in the sample. When porosity is held constant, increasing the thickness of the foam glass, the number of the apertures, and the thermal resistance can significantly improve the thermal insulation performance of the foam glass, which is consistent with real-life engineering applications. 

### 4.4. Effects of Porosity on Thermal Conductivity of Foam Glass

**Figure 9:**
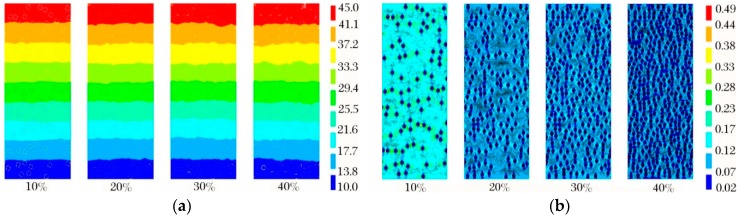
(**a**) Temperature nephogram; (**b**) Thermal vector nephogram.

The effect porosity has on thermal conductivity in the foam glass was investigated. Experiments were performed on samples with an aperture of 0.8 mm with varying porosity (10%, 20%, 30% and 40%); results from these experiments are shown in Figure 9.

From Figure 9a, we observe that with the increase of sample porosity, the temperature range within the material is essentially unchanged. The temperature fluctuation at the boundary, however, becomes significantly larger, indicating that the temperature distribution of the sample is greatly affected by the number of apertures. From Figure 9b, it is shown that the thermal vector distribution of samples with varying thickness remains constant, indicating that more uniformly distributed and orderly arranged apertures in the foam glass material results in a greater thermal resistance, and accordingly improves the thermal insulation of the material.

**Figure 10:**
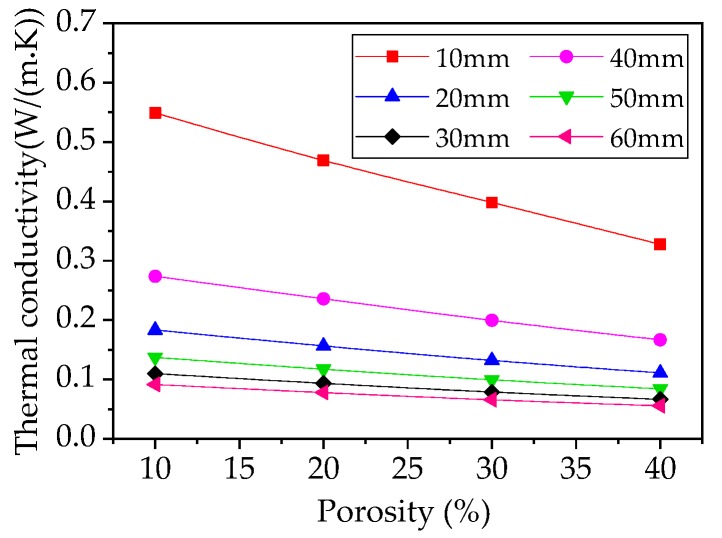
Relationship between porosity and thermal conductivity of samples at different thicknesses.

Lastly, the relationship between the porosity and the thermal conductivity of the samples with different thickness (10 mm, 20 mm, 30 mm, 40 mm, 50 mm, and 60 mm) is shown in Figure 10.

Clearly, when the thickness of the sample is constant, thermal conductivity is linearly proportional to porosity: as porosity increases, the thermal conductivity gradually decreases. When the thickness of the sample increases, the relationship curve between thermal conductivity and porosity tends to be flat, thereby indicating that the thicker the material, the weaker the impact porosity has on the thermal insulation of the material.

## 5. Conclusions

The percentage of the apertures of the samples is approximately a normal distribution with change of the average aperture size. When the porosity is held constant, the thermal conductivity decreases overall with increasing aperture, and when porosity is varied, the thermal conductivity decreases significantly with increasing porosity, therefore, the thermal insulation of the foam glass material is directly influenced by the porosity.

The apertures in the foam glass samples can significantly block the heat flow transmission. When the heat flow meets the apertures, it diverts to the sides of the aperture rapidly, transmitting along the back side of the aperture wall. Almost no heat flows into the apertures and thus the internal heat flow is minor. The heat flow between apertures gradually decreases when the porosity of the sample increases. With increasing porosity, the thermal resistance of the foam glass sample increases when the thickness of the sample is constant, thus the thermal insulation of the material is improved.

The distribution characteristics of the apertures directly determine the length and number of thermal resistance bands in the sample, which in turn exemplifies the significance it has on the thermal insulation of foam glass materials. The more uniformly distributed and orderly arranged the apertures were in the foam glass materials, the greater the thermal resistance of the material, and accordingly the better the thermal insulation.

## Figures and Tables

**Table 1:** The chemical composition and physical indicators of the fly ash and glass powder.

Designation	Mass Fraction (%)						Fineness (%)	Loss on Ignition (%)
	SiO_2_	Al_2_O_3_	Fe_2_O_3_	CaO	MgO	K_2_O+Na_2_O		
Fly ash	59.84	30.77	3.30	1.84	2.35	1.90	4.70	4.90
Waste glass	72.33	1.40	0.15	8.62	4.72	12.78	-	-

**Table 2:** The L_9_(3^4^) Orthogonal test factors and levels.

	Factors	ω (%)		Foaming Temperature (°C)	Foaming Time (min)
Level		Fly Ash	Na_2_CO_3_		
1		20	2	850	20
2		25	4	860	25
3		30	6	870	30

Note: ω is mass fraction.

**Table 3:** The L_9_(3^4^) Orthogonal experiment results.

No.	F (%)	C (%)	T (°C)	S (min)	Thermal Conductivity (w/(m·k))	Porosity (%)
Z1	20	2	850	20	0.0587	30.54
Z2	20	4	860	25	0.0569	41.29
Z3	20	6	870	30	0.0545	44.29
Z4	25	2	860	30	0.0671	28.64
Z5	25	4	870	20	0.0570	50.94
Z6	25	6	850	25	0.0608	45.67
Z7	30	2	870	25	0.0656	43.00
Z8	30	4	850	30	0.0675	36.69
Z9	30	6	860	20	0.0703	36.67

Note: F, C, T and S are fly ash, sodium carbonate, foaming temperature and foaming time, respectively.

**Table 4:** The results of statistical analysis of the aperture in foam glass.

Sample No.			Z1	Z2	Z3	Z4	Z5	Z6	Z7	Z8	Z9
Aperture number	1	D ≥ 3 mm	1	1	1	2	1	2	1	2	0
	2	2 mm ≤ d < 3 mm	3	2	2	3	2	3	1	1	3
	3	1 mm ≤ d < 2 mm	23	20	25	23	27	24	20	23	20
	4	0.5 mm ≤ d < 1 mm	38	43	33	45	34	45	46	38	36
	5	0.1 mm ≤ d < 0.5 mm	28	32	25	15	25	13	11	17	25
	6	0.01 mm ≤ d < 0.1 mm	1	1	1	1	1	1	1	1	1
summation			94	99	87	89	90	88	80	82	85
Average aperture/mm			0.65	0.64	0.67	0.57	0.65	0.62	0.61	0.81	0.60

**Table 5:** Comparison of simulated and measured values of thermal conductivity of foam glass.

Material	Thermal Conductivity Simulated Value (W/(m·K))	Thermal Conductivity Measured Value (W/(m·K))	Relative Error (%)
Foam glass	0.0612	0.0587	4.08
